# Association between aberrant amino acid metabolism and nonchromosomal modifications fetal structural anomalies: A cohort study

**DOI:** 10.3389/fendo.2023.1072461

**Published:** 2023-02-24

**Authors:** Huizhen Yuan, Chang Liu, Xinrong Wang, Tingting Huang, Danping Liu, Shuhui Huang, Zeming Wu, Yanqiu Liu, Peiyuan Yin, Bicheng Yang

**Affiliations:** ^1^ Jiangxi Key Laboratory of Birth Defect Prevention and Control, Jiangxi Maternal and Child Health Hospital, Nanchang, China; ^2^ Chinese Academy of Sciences Key Laboratory of Separation Sciences for Analytical Chemistry, National Chromatographic R&A Center, Dalian Institute of Chemical Physics, Chinese Academy of Sciences, Dalian, China; ^3^ Key Laboratory of Integrative Medicine, The First Affiliated Hospital of Dalian Medical University, Dalian, China; ^4^ Institute of Integrative Medicine, Dalian Medical University, Dalian, China; ^5^ iPhenome Biotechnology (Yun Pu Kang) Inc., Dalian, China

**Keywords:** fetal structural anomalies, amniotic fluid, metabolic, maternal, pregnancy, amino acid

## Abstract

**Background:**

More than half of the cases of fetal structural anomalies have no known cause with standard investigations like karyotype testing and chromosomal microarray. The differential metabolic profiles of amniotic fluid (AF) and maternal blood may reveal valuable information about the physiological processes of fetal development, which may provide valuable biomarkers for fetal health diagnostics.

**Methods:**

This cohort study of singleton-pregnant women had indications for amniocentesis, including structural anomalies and a positive result from maternal serum screening or non-invasive prenatal testing, but did not have any positive abnormal karyotype or chromosomal microarray analysis results. A total of 1580 participants were enrolled between June 2021 and March 2022. Of the 1580 pregnant women who underwent amniocentesis, 294 were included in the analysis. There were 137 pregnant women in the discovery cohort and 157 in the validation cohort.

**Results:**

High-coverage untargeted metabolomic analysis of AF revealed distinct metabolic signatures with 321 of the 602 metabolites measured (53%) (false discovery rate, q < 0.005), among which amino acids predominantly changed in structural anomalies. Targeted metabolomics identified glutamate and glutamine as novel predictive markers for structural anomalies, their vital role was also confirmed in the validation cohort with great predictive ability, and the area under the receiver operating characteristic curves (AUCs) were 0.862 and 0.894 respectively. And AUCs for glutamine/glutamate were 0.913 and 0.903 among the two cohorts.

**Conclusions:**

Our results suggested that the aberrant glutamine/glutamate metabolism in AF is associated with nonchromosomal modificantions fetal structural anomalies. Based on our findings, a novel screening method could be established for the nonchromosomal modificantions fetal structural anomalies. And the results also indicate that monitoring fetal metabolic conditions (especially glutamine and glutamine metabolism) may be helpful for antenatal diagnosis and therapy.

## Graphical Abstract

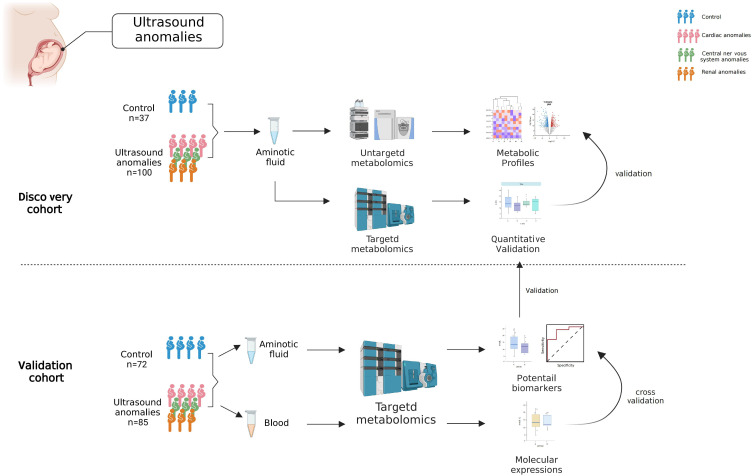

## Introduction

Fetal structural anomalies, which can range from minor deficiencies in a single organ to severe multi-organ system malformations, have a considerable impact on fetal morbidity and mortality (1). Prenatal ultrasound is now regarded as a routine analysis in obstetrical care, and with increasingly high resolution, fetal structural anomalies are identified in approximately 3% of pregnancies. Fetal structural anomalies have various genetic causes, including chromosomal aneuploidy, copy number variations (CNVs), and pathogenic sequence variants in developmental genes (2). Genetic investigations are essential for the assessment and clinical triage of fetal structural anomalies. Clinically, when fetal anomalies are identified, further prospective evaluations included karyotype testing and chromosomal microarray analysis (CMA) to detect aneuploidies and CNVs (3, 4). Overall, approximately 32% of fetuses with a structural anomaly identified by ultrasound have a clinically relevant abnormal karyotype, and 6.5% of them have a causative CNV (1, 3–5). Additionally, where karyotype testing and CMA failed to determine the underlying cause, whole-exome sequencing was reported to identify a well-described genetic cause in 8.5-10% of fetuses with structural anomalies (2, 6). However, more than 50% of fetal structural anomalies are left without a prospectively screening or identification method.

Pregnancy is related to the onset of many adaptation processes that change throughout gestation (7). Maternal blood constantly exchanges with the fetus’s blood through the placenta to provide the nutrients needed for fetal growth and development. Amniotic fluid (AF) can also be considered a pool of metabolites reflecting the biological process of anabolism and catabolism (8, 9). The biochemical nature of AF and maternal blood makes them extremely valuable materials for fetal health diagnostics.

Spurred by tremendous technological advancements, the metabolome has become widely acknowledged as the dynamic and sensitive expression of biological phenotypes at the molecular level, placing metabolomics at the forefront of biomarker and mechanistic discoveries associated with pathophysiological processes (10). Untargeted metabolomics is applied to measure the most comprehensive range of compounds or putative metabolites present in an extracted sample without prior knowledge of the metabolome (11). In contrast, targeted metabolomics focuses on a small group (50–500) of compounds of interest; here, methods are generated and optimized for the investigation of specific metabolites and metabolic pathways with higher sensitivity and selectivity than untargeted metabolomics (12). The targeted analysis is also outstanding for hypothesis validation and expanding upon the results of untargeted analysis (13).

Liquid chromatography-tandem mass spectrometry (LC-MS/MS) is a current, routine, highly accurate application in newborn screening (14, 15). Similarly, metabolomics can be applied to fetal malformations by exploring the AF metabolome, and several studies have reported promising results (16, 17), revealing the possibility of using this technology in clinical practice. Since AF can reflect both maternal and fetal health, linking AF metabolic profiles with structural anomalies is conducive to biomarker discovery, and will better guide clinical practice.

The present study aimed to characterize the metabolic signature of AF in fetal structural anomalies. Also, we tried to investigate whether metabolic changes reflect maternal or fetal conditions. In this study, we measured AF metabolites in the structural anomalies and control groups from two independent cohorts using both untargeted and targeted metabolomics. First, a high-coverage untargeted metabolomic assay based on ultra-high performance liquid chromatography-tandem mass spectrometry (UHPLC-MS/MS) was applied to 137 participants (the discovery cohort). To assay the changes in metabolites more quantitatively, we performed targeted metabolomic analysis using the UHPLC-MS/MS system and isotope-labeled internal standards. The findings in the discovery cohort were confirmed by targeted metabolomic analysis of a validation cohort of 157 participants. At the same time, we analyzed maternal serum metabolites using targeted metabolomics, which reflected the amino acid metabolism of the mothers.

## Materials and methods

### Study design and participant enrollment

This study was approved by the medical ethics committee of Jiangxi Maternal and Child Health Hospital (Approval number: EC-KT-202210). All the participants provided written informed consent. All participants were recruited from the prenatal diagnosis center of Jiangxi Maternal and Child Health Hospital from June 2021 to March 2022. Inclusion criterion: Pregnant women who had an indication for amniocentesis, including structural anomalies and a positive result from maternal serum screening or non-invasive prenatal testing. Exclusion criteria: (1) abnormal karyotype or chromosomal microarray analysis results; gestational age beyond 140-154 days; (3) multiple pregnancies; (4) other risk factors for prenatal diagnoses. Finally, 294 participants were included and separated into the discovery (n= 137, from June 2021 to October 2021) and validation (n= 157, from November 2021 to March 2022) cohorts. Fetuses with structural anomalies were categorized into three phenotypic groups based on abnormalities in different organ systems detected by ultrasound, including cardiac, central nervous systems, and renal anomalies. The control group in this study included women with singleton pregnancies whose fetuses had no structural malformations, but who had indications for amniocentesis, including a positive result from maternal serum screening or non-invasive prenatal testing.

### Collection and processing of samples

20-25 mL of AF and 3-5 mL of blood were obtained from the pregnant women at the time of amniocentesis. The AF was centrifuged at 1200 rpm for 10 min at 4°C, and the supernatant was collected. Blood was placed at 4°C for 1 h and centrifuged at 3000 rpm at 4°C for 10 min, and serum was collected from the upper layer. All samples were stored at -80°C before analysis, and their use for research was approved by the ethical committee. In the validation cohort, AF and blood samples were obtained from the same pregnant woman.

### Untargeted LC-MS metabolomics profiling

Broad-based metabolomic profiling was performed using UHPLC-MS/MS platform. Further details are provided in the **Supplementary Material**.

### Targeted LC-MS metabolomics data collection and processing

Fifty-four amino acids and their derivatives were quantified using a Shimadzu LC-20ADXR (Shimadzu, Kyoto, Japan) coupled with a Sciex 5500+ triple quadrupole mass spectrometer (AB Sciex, Singapore). Further details are provided in the **Supplementary Material**.

### Statistical analysis

The metabolites included in the statistical analyses were those which were consistently detected in at least 80% of the samples. The metabolome data derived from different methods were normalized. Data scaling was assessed using Pareto scaling. Multivariate statistical analyses, partial least squares discrimination (PLS-DA), functional enrichment, metabolic pathway analysis of metabolites and lipids, and receiver operating characteristic (ROC) analysis, and the respective area under the ROC curve (AUC) were performed using an online data analysis platform- MetaboAnalyst 5.0 (https://www.metaboanalyst.ca). Unit statistical analyses, such as t-tests, were performed using SPSS software (version 26.0; IBM, USA). Bar and line plots were drawn by GraphPad Prism 8.0 (GraphPad Software Inc., USA). Chemical similarity enrichment analysis was conducted using ChemRICH R package (18), and significant metabolites alterations were visualized in an enhanced heat map in gplots package using the in R (version 3.6). All p-values involved in this study were two-tailed probabilities and were adjusted by false discovery rate (FDR). Differences were considered statistically significant at FDR <0.05.

## Results

### High-coverage untargeted metabolomics analysis revealed distinct metabolic signatures with amino acids predominantly changed in the structural anomalies group

**Figure 1:**
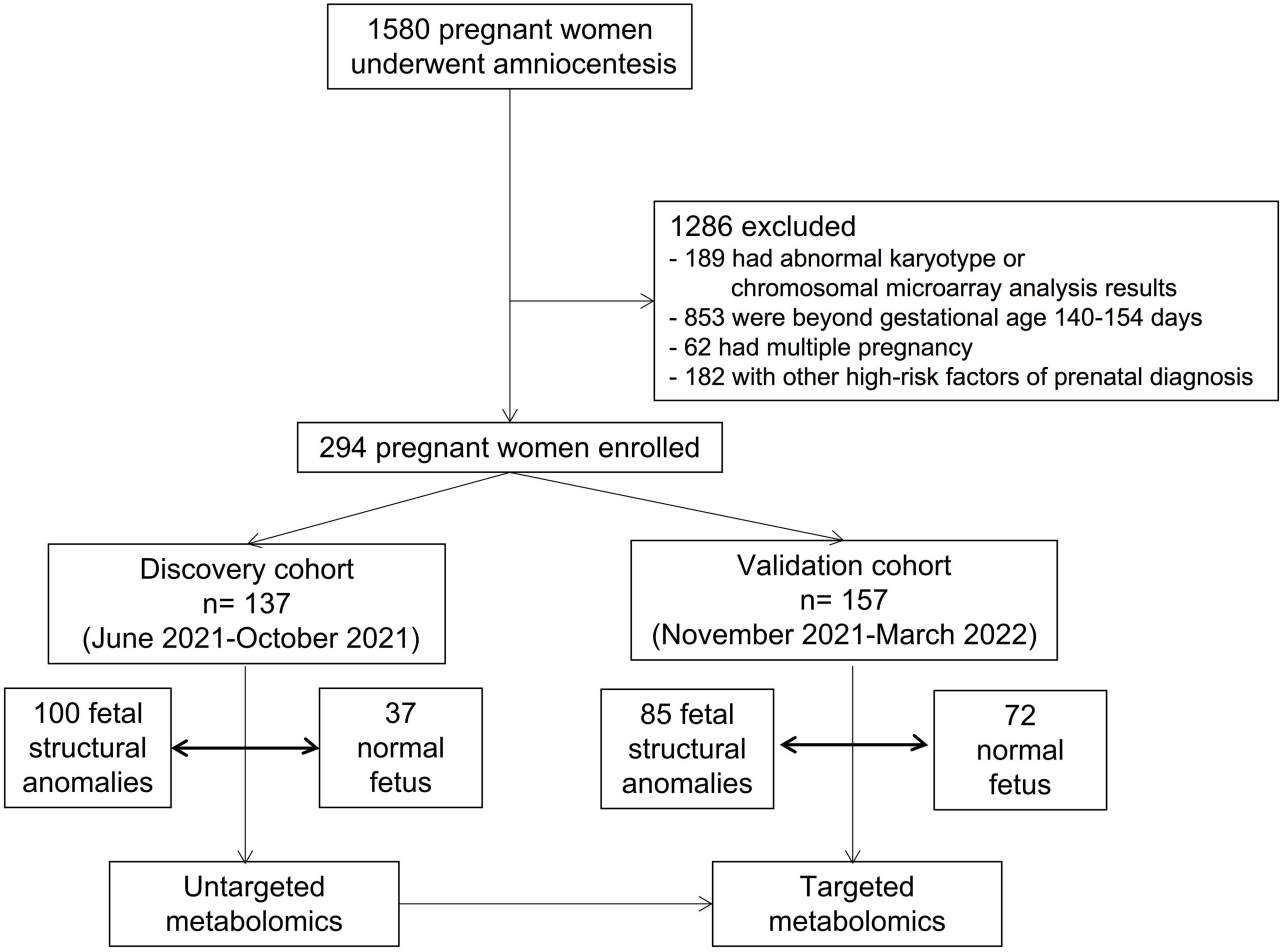
Study outline of workflow.

**Figure 2:**
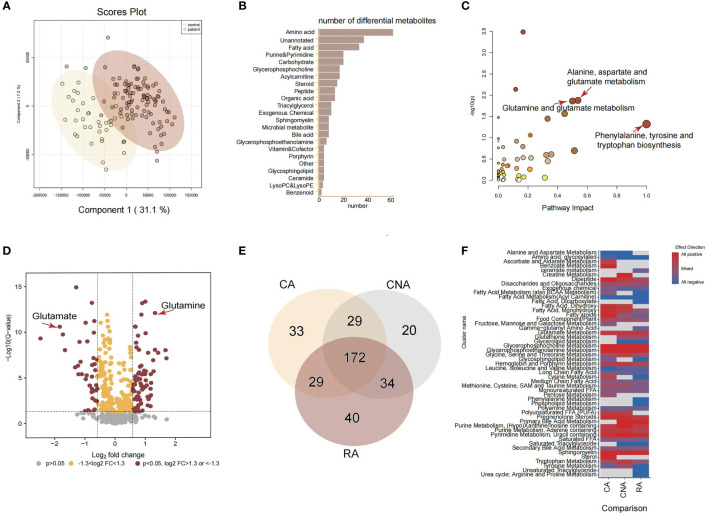
Amniotic fluid metabolic landscape for fetal ultrasound anomalies. **(A)** PLS-DA score plot for untargeted metabolomics data. **(B)** Classed enrichment analysis for differential metabolites between ultrasound anomalies group and the control group. **(C)** Pathway enrichment analysis for significantly different metabolites between ultrasound anomalies group and the control group. **(D)** Volcano plot for all metabolites from untargeted metabolomics. **(E)** Venn plot of differential metabolites from three kinds of ultrasound anomalies compared with the control group. **(F)** Chemical similarity enrichment analysis for differential metabolites from three kinds of ultrasound anomalies. CA, cardiac anomalies; CNA, central nervous system anomalies; RA, renal anomalies.

To comprehensively detect the metabolic profiles of structural anomalies, we implemented a high-coverage untargeted metabolomic analysis of AF samples by integrating five different analytical methods that could cover both hydrophobic and hydrophilic metabolomes. Between June 2021 and March 2022, 1580 pregnant women whose fetuses were diagnosed with structural anomalies were screened for their eligibility for inclusion in our study (**Figure 1**). Finally, 137 and 157 participants prospectively enrolled in the study as described in **Table 1**. The untargeted metabolomic analysis enabled the detection and relative quantification of 602 metabolites in all AF samples. As shown in the PLS-DA score plot, the structural anomalies group was separated from the control group in the direction of the first principal component (**Figure 2A**). Running 10-fold cross-validation showed that the accuracy of one component was 0.87 (0.54 for R2 and 0.50 for Q2) (**Supplementary Table 1**). Moreover, 321 metabolites were identified as differential metabolites between the structural anomalies and control groups (FDR, q <0.05) (**Supplementary Table 2**, **Supplementary Figure 1A**). Among these, differential amino acids were the most abundant (**Figure 2B**). The KEGG pathway enrichment analysis of these differential metabolites also showed that amino acid metabolic pathways, such as glutamine (Gln) and glutamate (Glu) metabolism; alanine, aspartate and Glu metabolism; and phenylalanine, tyrosine and tryptophan biosynthesis, were the most significant changed (**Figure 2C**). Among all amino acids, Gln (32% increase, FDR, q<1×10−13) and Glu (84% decrease, FDR, q<1×10−11) were the significantly the significantly altered metabolite in structural anomalies group (**Figure 2D**, **Supplementary Table 2**).

**Table 1:** Characteristics of cases with structural anomalies and matched controls.

	Structural anomalies		Control	
	Discovery cohort	Validation cohort	Discovery cohort	Validation cohort
**AF samples, No.(%)**	100 (34%)	85 (29%)	37 (13%)	72 (24%)
**blood samples,No.(%)**	0	85 (54%)	0	72 (56%)
**gestational age when underwent amniocentesis (days)**	146.76 ± 4.82	147.14 ± 4.61	145.82 ± 3.96	146.36 ± 4.68
**Maternal age (years)**	27.36 ± 2.12	25.68 ± 1.85	27.97 ± 2.32	29.07 ± 2.64
**Smoke during pregnancy**	0	0	0	0
**Fetal karyotype analysis results**	normal	normal	normal	normal
**Fetal chromosomal microarray analysis results**	normal	normal	normal	normal
**Maternal ethnicity**	Asian	Asian	Asian	Asian

Based on the above results, we focused on the amino acid changes among different structural anomalies, including cardiac, central nervous system, and renal system anomalies. Compared to the control group, each type of structural anomaly demonstrated a distinct metabolic profile, with 172 overlapping differential metabolites (**Figure 2E**). There were 14 amino acids in the 172 overlapping metabolites. Surprisingly, Glu levels were dramatically lower while Gln levels were significantly higher in the cardiac, central nervous system, and renal anomalies (**Supplementary Table 2**). Gln-Glu exchange is important in placental amino acid transport, and Gln and Glu are the most utilized amino acids in fetuses during late gestation. Therefore, we hypothesized that Gln and Glu are vital for the early diagnosis of fetal structural anomalies.

In addition to the significant changes in Glu metabolism in the three types of structural anomalies, it is worth noting that fetuses with renal anomalies uniquely showed significantly inhibited urea cycle (arginine and proline metabolism), and that creatine metabolism was positively regulated in fetuses with central nervous system anomalies subjects (**Figure 2F**). These metabolic pathway changes may be typical responses to different structural anomalies.

### Amniotic fluid-targeted metabolomics of identified glutamate and glutamine as novel predictive markers for structural anomalies

We performed a targeted metabolomic assay of 54 amino acids and their derivatives to quantify the metabolite changes in the structural anomalies and control groups more precisely. We first quantified AF amino acids obtained from 137 participants in the discovery cohort, confirming that aberrant amino acid metabolism occurred in the structural anomalies group (**Supplementary Table 3**). Gln and Glu were significantly altered in targeted metabolomics.

**Figure 3:**
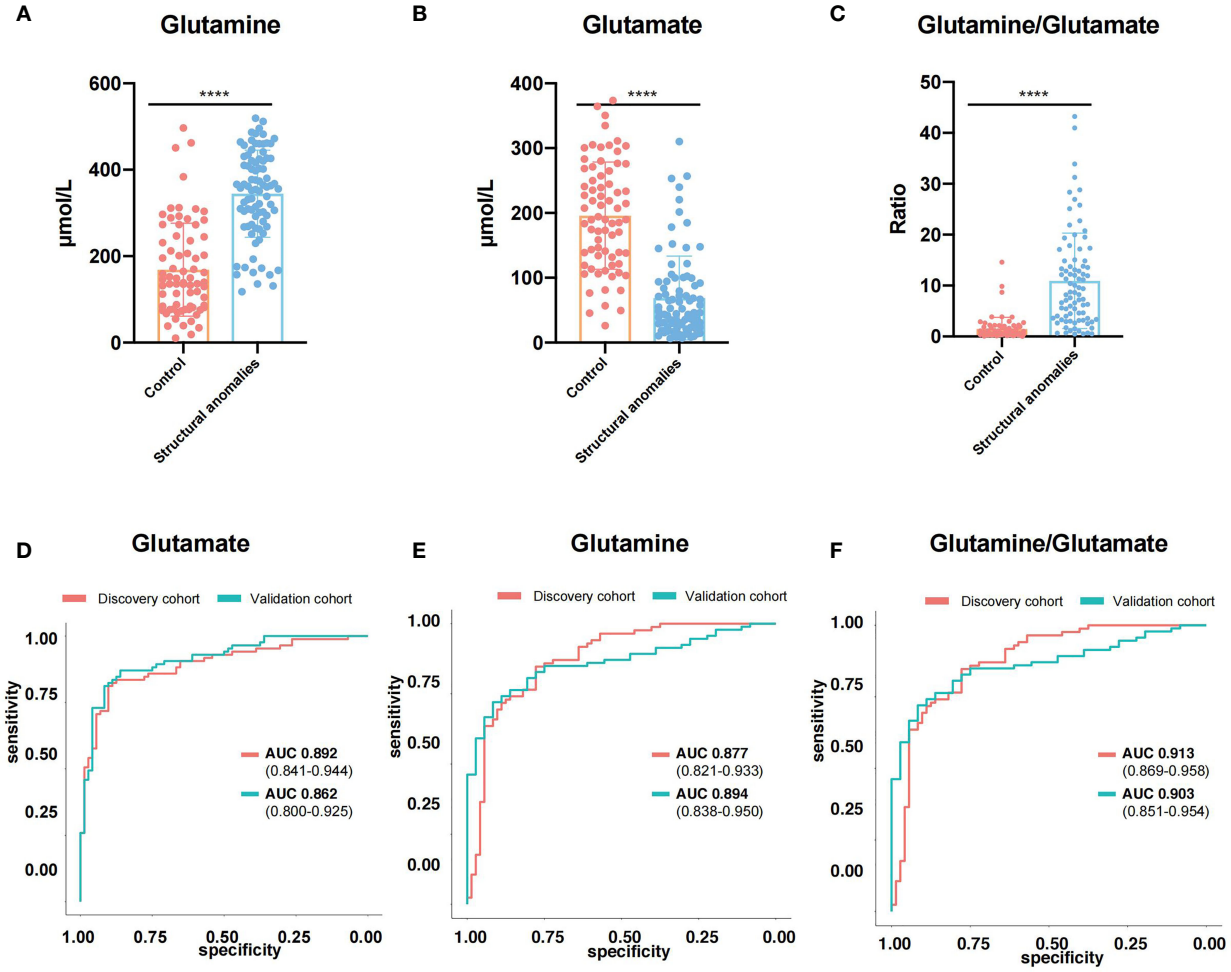
Glutamine and Glutamate were novel predictive markers for ultrasound anomalies. **(A–C)** Expression of glutamine **(A)**, glutamate **(B)** and glutamine/glutamate **(C)** in amniotic fluid of validation cohort. **(D, E)** ROC curves of glutamine **(D)**, glutamate **(E)** and glutamine/glutamate **(F)** in discovery cohort (red line) and validation cohort (blue line). ****, P<0.0001.

To further validated these results, we applied targeted metabolomics to the validation cohort. Based on the concentrations presented in the different groups, 33 amino acids, including Gln and Glu, showed significant differences between the structural anomalies and control groups (**Supplementary Table 4**). Twenty amino acids were shared by the three types of structural anomalies (cardiac, central nervous system and renal anomalies), and significant differences existed between the structural anomalies and control groups (**Supplementary Figures 1B, C**). We found that Glu levels in the AF were significantly lower (**Figure 3A**), while Gln levels were significantly higher in the structural anomalies group than in the control group (**Figure 3B**). Using Gln/Glu as a metric indicating Gln-Glu conversion, we found that this ratio fell approximately 14-fold on an average among participants in the structural anomalies group (**Figure 3C**). Notably, regardless of the types of anomaly present, the Gln/Glu ratio was significantly reduced in the structural anomalies group than in the control group (**Supplementary Figure 1D**). These results were consistent with our findings in the discovery cohort (**Supplementary Figure 1E**). In addition, Glu (AUC=0.862, 95%CI: 0.800-0.925), Gln (AUC=0.894, 95%CI: 0.838-0.950) and Gln/Glu (AUC=0.903, 95%CI: 0.851-0.954) had great prediction ability in distinguishing structural anomalies from the control group in the validation cohort (**Figure 3D–F**).

**Figure 4:**
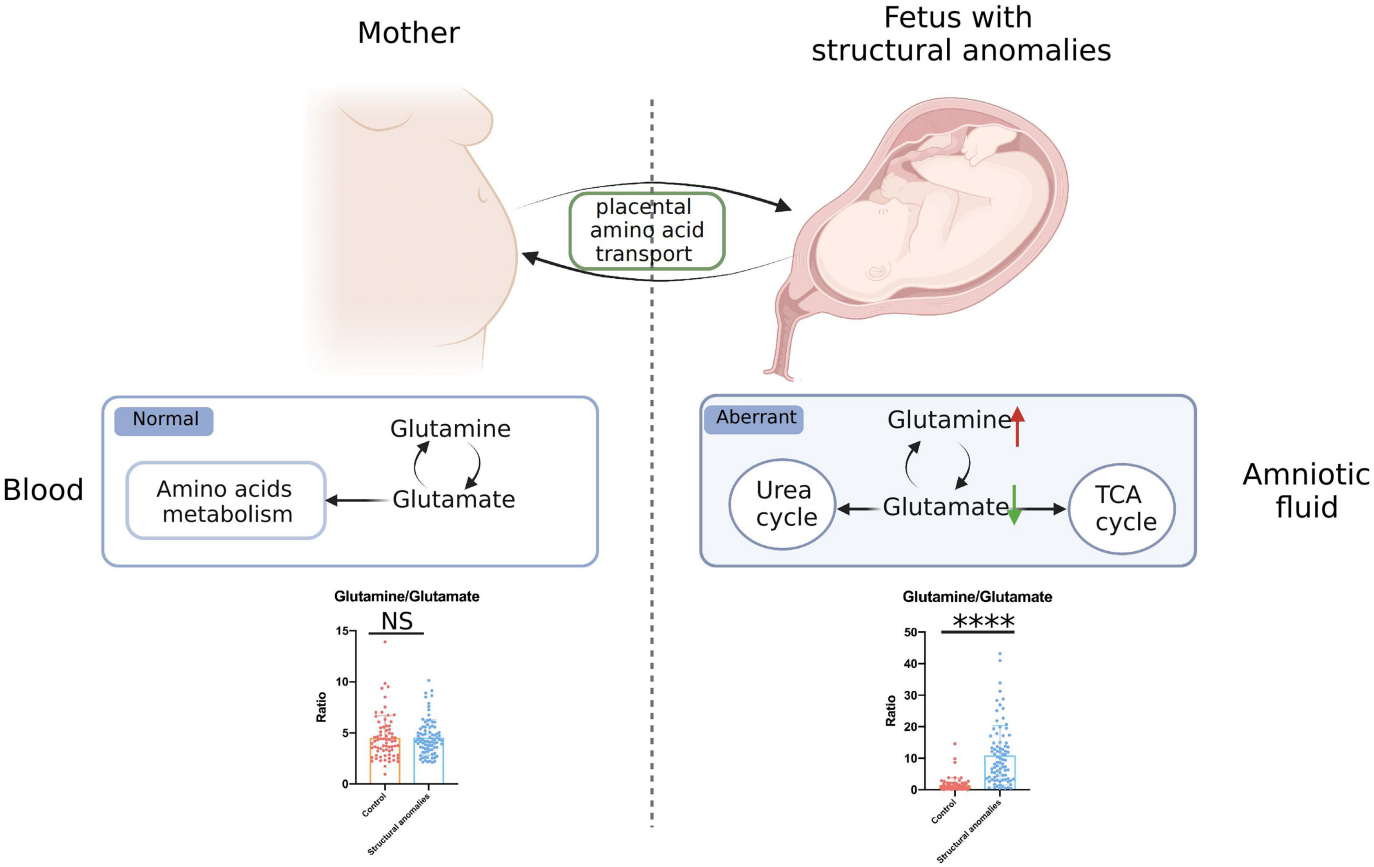
General view of aberrant glutamate and glutamine metabolism in fetal ultrasound anomalies. NS, no significance; ****, P<0.0001.

We then investigated whether the Gln/Glu in AF correlated with maternal metabolic conditions. Serum samples were collected from women in the validation cohort and analyzed using the same amino acid-targeted metabolomic assay. Notably, maternal serum Glu (**Supplementary Figure 1F**) and Gln levels (**Supplementary Figure 1G**) did not differ significantly between the structural anomalies and control groups. Gln/Glu ratio also did not differ between the two groups (**Supplementary Figure 1H**). In addition, almost all the quantified amino acids demonstrated no big differences between the structural anomalies group and control group (**Supplementary Table 5**), except for threonine (**Supplementary Figure 1I**) and leucyl-leucine (**Supplementary Figure 1J**). Taken together, these results suggest that changes in Gln/Glu ratio in the AF of the structural anomalies group are associated with the fetal condition rather than the maternal condition (**Figure 4**).

## Discussion

Despite the use of karyotype testing and chromosomal microarray as routine investigations in obstetric care, a large proportion of fetal structural anomalies still have no proven cause. Herein, we explored the underlying causes of fetal malformations using AF metabolomics study. First, we performed an untargeted metabolomic assay on AF samples, starting with the discovery cohort. The results demonstrated that AF metabolic signatures were remarkably altered in the structural anomalies group compared to the control group. The most apparent alterations were observed in amino acids and their derivatives. These amino acid changes were further confirmed using targeted metabolomics, and we found 23 amino acids that were differentially expressed in the three types of structural anomalies (cardiac, central nervous system, and renal anomalies). Among these amino acids, Glu and Gln were the most significantly altered metabolites. The structural anomalies group was characterized by a significantly lower Gln/Glu ratio than the control group. To strengthen this finding, the results were validated using samples from an independent validation cohort. The results of the validation cohort were consistent with those of the discovery cohort; aberrant Glu and Gln metabolism was found in fetal structural anomalies. In addition, analysis of maternal blood samples through targeted metabolomics demonstrated no significant difference in Gln/Glu ratio between the fetal structural anomalies and the control groups, suggesting that the contributors to these Glu-Gln changes in AF were closely related to fetal metabolic conditions rather than maternal metabolic status. It is also worth noting that most amino acids in maternal blood did not show significant changes in the structural anomalies and the control groups.

During pregnancy, amino acids serve as important precursors for the biosynthesis of macromolecules, including proteins and nucleotides, which are involved in fetal development and growth (19–21). Glu and Gln are among the most abundant and most utilized amino acids in the fetus during late pregnancy (19). The human placenta mediates the net transfer of amino acids to the fetus, with amino acid concentrations being generally higher in the fetus than in the mother, indicating an active transfer process across the placenta (22, 23). One notable exception to this process is Glu, which is the net placental uptake from the fetus (23). To meet the acquisitive demand for nutrients, Gln, a non-essential amino acid, is essential when fetal demand for amino acids exceeds maternal supply during pregnancy (24, 25). This demand is met through the interorgan recycling of Gln and Glu. In the fetal liver, the deamination of Gln produces Glu. Glu is transported across the syncytiotrophoblast microvillous membrane and basal membranes by high-affinity excitatory amino acid transporters and is converted to Gln in the placenta (26, 27). Glu is also an important nitrogen resource and a precursor of γ-aminobutyric acid, a key inhibitory neurotransmitter (28, 29). Therefore, the Glu-Gln cycle and exchange in the placenta-fetus unit likely play important roles in fetal growth and development.

In our study, the significant increase in Gln/Glu ratio in the AF observed in the fetal structural anomalies group suggested a disturbing Glu-Gln cycle in the fetus rather than in the mother, since no obvious changes were detected for either Glu or Gln in maternal blood. Decreased levels of Glu and increased levels of Gln in AF have also been reported in the studies of fetal malformations, prediagnostic gestational diabetes, preterm delivery and early rupture of membranes (16, 30). The underlying cause may be the dysfunction of transporters utilized by Glu and Gln. The amino acids that the fetus requires for metabolic processes and biosynthesis pathways can only be obtained from the placenta and delivered by different amino acid transporters (23, 31). For example, in fetal growth restriction, the initial rate of uptake of Gln and Glu into placental villous fragments is reportedly reduced but increases with the expression of their transporter proteins (Gln: LAT1, LAT2, SNAT5, Glu: EAAT1) (32, 33). Transporter activity is not simply determined by the protein expression levels; it is also influenced by factors that regulate substrate levels on both sides of the membrane. Interestingly, a study demonstrated that Glu efflux down its transmembrane gradient drives placental uptake *via* OAT4 and OATP2B1 from the fetal circulation and that the reuptake of Glu maintains this driving gradient, although OAT4 and OATP2B1 are not currently understood Glu transporters (26).

In the group with renal anomalies, we also found inhibited urea cycle metabolism. Arginine is the precursor for the synthesis of ornithine, proline, and nitric oxide (34), detecting the levels of arginine and its metabolites may provide insight into discriminating fetal renal anomalies and monitoring fetal urinary development.

However, there are some limitations to our study. First, this study was limited to one center: the Prenatal Diagnosis Center of Jiangxi Maternal and Child Health Hospital. UHPLC-MS/MS analysis is simple and sensitive, and it uses only a small amount of AF for metabolic analysis, AF acquisition is still invasive. Additionally, details of clinical examination results were not available in our study, so the study did not reveal the correlations between changed metabolites and the clinical data.

## Data availability statement

The original contributions presented in the study are included in the article/**Supplementary Material**. Further inquiries can be directed to the corresponding authors.

## Ethics statement

The studies involving human participants were reviewed and approved by The medical ethics committee of Jiangxi Maternal and Child Health Hospital. The patients/participants provided their written informed consent to participate in this study. Written informed consent was obtained from the individual(s) for the publication of any potentially identifiable images or data included in this article.

## Author contributions

BY, YL, PY conceived and designed the experiment. HY, XW, TH,DL and SH collected the samples and clinical information. HY, CL, ZW performed the experiments and analyzed the data. CL, ZW drafted the manuscript, BY, YL, PY reviewed the manuscript, and BY, PY edited the final manuscript. All authors contributed to the article and approved the submitted version.
