# Supplementary material for: Association between aberrant amino acid metabolism and nonchromosomal modifications fetal structural anomalies: A cohort study
Source: Front Endocrinol (Lausanne). 2023 Feb 24;14:1072461. doi: 10.3389/fendo.2023.1072461 (PMC9998993; doi:10.3389/fendo.2023.1072461)
Supplement: Supplementary file 2 [file DataSheet_2.docx]

Supplementary Material

# Supplementary Data

**Untargeted LC-MS metabolomics data collection and processing**

For untargeted metabolomics, polar extracts of AF samples were thawed at room temperature. 150 µL AF was placed in a centrifuge tube with 600 µL of methanol (Fisher Scientific, Fair Lawn, USA) and vortexed for 3 min (1000 rpm). Then, 900 µL MTBE (Sigma-Aldrich, St. Louis, USA), and 250 L water purified with Milli-Q system (Merck KGaA, Darmstadt, Germany) was added and vortexed for 10 min. The mixture was kept at 4 °C for 10 min to facilitate stratification, followed by centrifugation at 13000 g for 15 min at 4 °C. After stratification, 200 µL of the polar extract was transferred from the lower layer and placed in a new centrifuge tube. For lipid extracts, 20 µL AF was placed in a centrifuge tube with 120 µL of methanol (Fisher Scientific, Fair Lawn, USA) and vortexed for 3 min (1000 rpm). Then, 360 µL MTBE (Sigma-Aldrich, St. Louis, USA), and 100 L Milli-Q (Merck KGaA, Darmstadt, Germany) purified water was added and vortexed for 10 min. The mixture was kept at 4 °C for 10 min to facilitate stratification, followed by centrifugation at 13000 × g for 15 min at 4 °C. After stratification, 200 µL of the lipid extract was transferred from the lower layer and placed in a new centrifuge tube.

The remaining extracts in each sample tube were all mixed and oscillated evenly. At 10,000 × g for 10 min, 200 µL of the lipid layer and 200 µL of the polar layer were placed in two new 2 mL centrifuge tubes and used as quality control (QC) samples. Finally, all samples were concentrated and dried by vacuum centrifugation.

For targeted metabolomics, transfer 50μL AF or serum samples, different concentrations of standard curve to 750 μL 96-well plate (Waters Corporation, Milford, USA); 10 μL of internal standard mixed standard amino acid internal standard and 200 μL of extraction reagent were added, followed by 1500 rpm × 3 min shaking, then centrifuged the tubes at 5300 RPM for 20 min, finally transferred 130 μL supernatant to 450 μL tip bottom 96-well plate.

Add 130 μL ultra-pure water for dilution, swirled the plate at 1500 rpm for 3 min, then centrifuged at 5300 rpm for 20 min. Finally, transferred 200 μL supernatant to another 450 μL tip bottom 96-well plate for targeted metabolomics analysis.

We adopted untargeted metabolomic method (meta-PhenotyperTM assay) published in previous report1. Briefly, three different analytical methods were used to profile polar metabolite extracts and another two methods for hydrophobic lipid extractswhich were all performed on an UltimateTM 3000 ultrahigh performance liquid chromatograph coupled with Q ExactiveTM quadrupole-Orbitrap high-resolution mass spectrometer (Thermo Scientific, San Jose, USA). Before analysis, polar extracts were accurately added to 100 µL acetonitrile-water complex solution (1:3, v/v), lipid extracts with 80 µL acetonitrile-isopropanol solution (1:1, v/v), vortexed for 5 min and centrifuged at 13,000 × g for 15 min at 4 °C. Take 90 µL supernatant of polar extracts and 70 µL supernatant of lipid extract for detection.

**Targeted LC-MS metabolomics data collection and processing**

The metabolites were chromatographically resolved on an Acquity UPLC BEH Amide column (1.7 μm, 2.1×100 mm) after 2 μL aliquots of metabolite extracts injected, and 90% acetonitrile in water as weak eluent and 50% acetonitrile in water as strong eluent which were both added ammonium formate and formic acid as buffer salt to improve separation. The chromatographic gradient ramped from 0% strong eluent to 70% in 12 min with 0.3 mL/min flowrate. The metabolites were ionized by a TurboVTM heated electrospray ionization source, and then detected by scheduled multiple reaction monitoring mode (MRM). The main parameters were optimized as follows: negative ionSpray voltage -4.5 kV; Curtain gas: 35 psig; Ion gas 1 and 2: 50 psig; heater temperature: 550℃; postive ionSpray voltage 5.5 kV; Curtain gas: 35 psi; Ion gas 1 and 2: 50 psi; heater temperature: 550℃.

# Supplementary Figures and Tables

## Supplementary Figures


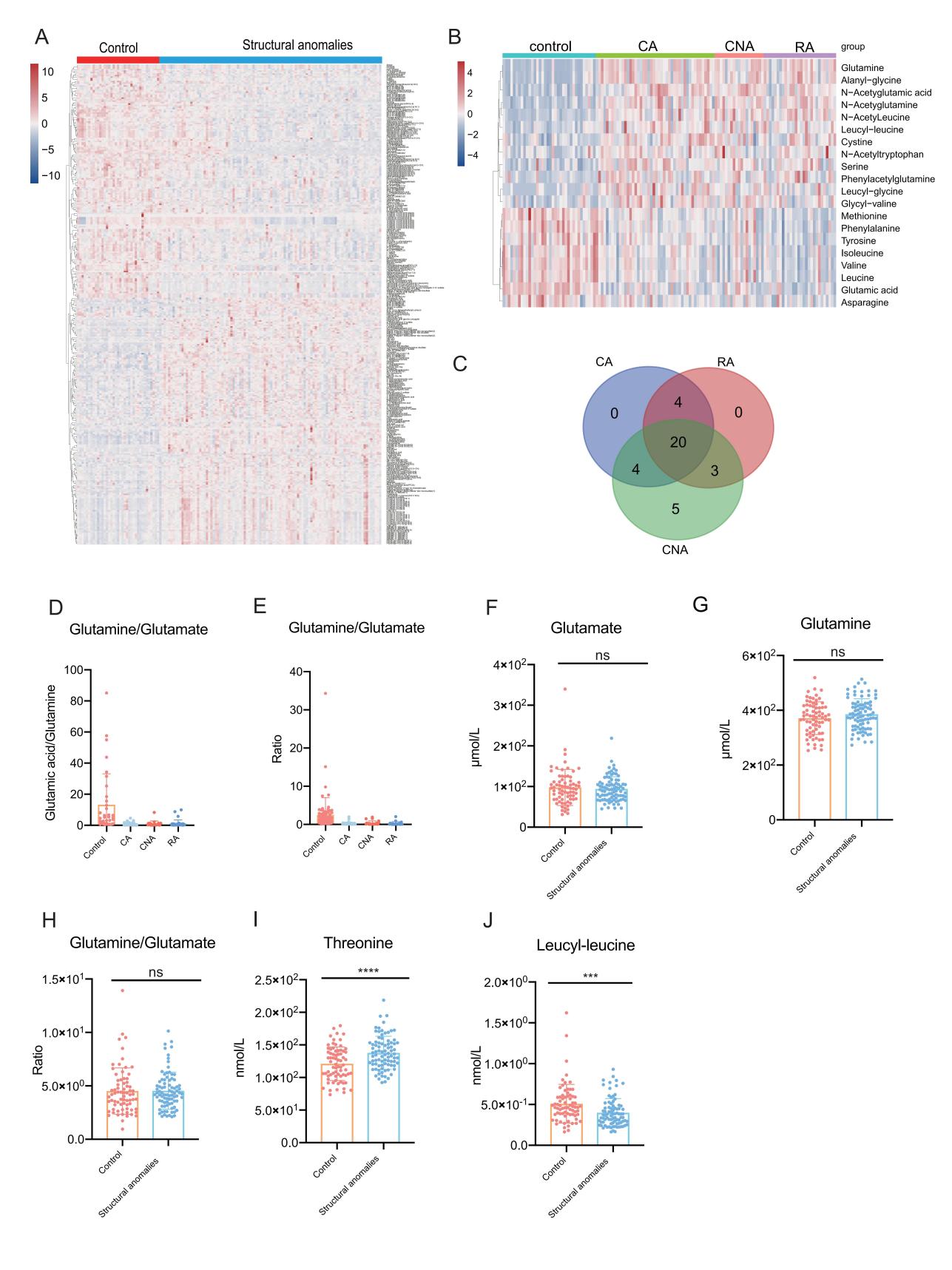


**Supplementary Figure 1.** (A) Heatmap showing the differential metabolites (FDR <0.05) between the structural anomalies and the control groups from untargeted metabolomics. (B) Heatmap showing the twenty amino acids shared by the three types of structural anomalies (cardiac, central nervous system and renal anomalies) from targeted metabolomics. (C) venn plot of overlapping amino acids among three types of anomalies. (D-E) glutamine/glutamate (Gln/Glu) ratio in the discovery cohort and validation cohort respectively. (F-J) expression of glutamate, glutamine, Gln/Glu, threonine and leucyl-leucine in maternal blood of the validation cohort. ****, p<0.0001; ***, p<0.001.
